# How Has COVID-19 Changed the Way We Do Virtual Care? A Scoping Review Protocol

**DOI:** 10.3390/healthcare10101847

**Published:** 2022-09-23

**Authors:** Cristina Catallo, Leinic Chung-Lee

**Affiliations:** Daphne Cockwell School of Nursing, Faculty of Community Services, Toronto Metropolitan University, Toronto, ON M5B 2K3, Canada

**Keywords:** virtual care, COVID-19, public health, communicable disease, pandemic, scoping review

## Abstract

The coronavirus disease (COVID-19) pandemic created worldwide interest and use of virtual care to support public health measures and reduce the spread of infection. While some forms of virtual care have been used prior to COVID-19 such as telemedicine, little is known about other virtual modalities such as video conferencing, wearables and other digital technologies. The COVID-19 pandemic has presented an opportunity to question the efficacy and safety of virtual care, especially in terms of patient outcomes among those self-isolating. The purpose of this scoping review is to examine the safety of virtual care among active COVID-19 patients in the community and examine the types and dose of virtual care. Finally, this review will examine what patient outcomes are identified from interventions delivered virtually to treat COVID-19. We followed a systematic process guided by the PRISMA checklist for scoping reviews with a comprehensive search strategy across four bibliographic databases and handsearching reference lists. We undertook a blinded, two-stage screening process with eligibility criteria. All citations and screening were managed using the DistillerSR software. Data were extracted using a data extraction tool developed for this project. The conclusions from this review will offer greater understanding for how virtual care can be used among community-based COVID-19 patients.

## 1. Introduction

The coronavirus disease (COVID-19) pandemic has changed the way we do virtual care. Public health measures and advice across the world have encouraged the public to “stay-at-home” and “self-isolate” if diagnosed with COVID-19. As a result, healthcare providers and organizations have quickly pivoted to provide outpatient care that was easily accessible to those isolating with COVID-19. Many organizations have since created virtual care programming delivered by a variety of healthcare providers for care at a distance, identifying potentially novel ways to offer healthcare. Virtual care has the potential to support the reduced spread of COVID-19 with a recent systematic review identifying that telehealth, a type of virtual care, can conserve health care resources and health system capacity [1]. There may be many optimum ways to utilize virtual care modalities, and much can be learned from the experiences of health care providers and patients throughout the pandemic. While we often think of virtual care as telehealth or telemedicine, the pandemic has encouraged healthcare providers to use different virtual modalities such as video conferencing, wearable devices, smartphone apps and other forms of digital technology. Existing literature suggests that there are benefits to both patient and the healthcare system from the use of telehealth, but we do not know if these benefits also extend to other types of virtual care modalities during times of public health emergency—such as a pandemic [1,2].

COVID-19 and the resulting restrictions have created a care gap in that patients who were recently diagnosed with COVID-19 and were often sent home from hospital to isolate but with no options for community-based follow-up [2]. This continues to be a gap in care and is exacerbated among those patients who are vulnerable for a variety of reasons. such as residing in low-income households, marginalized communities or who are elderly or socially isolated. Recent evidence suggests that vulnerable and marginalized patients may be at increased risk of COVID-19 infection and may have limited access to virtual care and new technologies or skills and resources. Because these patients are under-served, there is great potential that they will not receive or seek timely care [2,3]. Primary care providers are often a point of contact among those diagnosed with COVID-19 and who are actively isolating. From recent research in Canada, primary care office visits have decreased while virtual care visits increased for various health concerns during the height of the COVID-19 pandemic [4]. Virtual care can be ideal in terms of offering prompt and convenient healthcare to those patients who are unable to travel outside of their home [4,5] or for those with the financial and technological resources and skills to connect virtually or utilize newer digital innovations [4]. There are potential health system benefits, including reduced emergency department visits and hospitalization, greater ability to facilitate linkages across care sectors and the opportunity for increased patient and provider satisfaction [5]. Barriers to virtual care for health care providers include the inability to perform physical examinations, assess complex health issues, discuss sensitive information in a confidential setting and potentially miss information that would be visible during a face-to-face interaction [4]. While there is growing evidence about the use of virtual care in different health care settings, there is a need to better understand how virtual care, beyond just telephone-based care, has been used among COVID-19 newly diagnosed patients who are isolating in place. This includes the contextual understanding for the types of virtual care modalities offered to patients, how they have been utilized and how well they work for treating isolating COVID-19 patients. The goal of this scoping review is to examine the nature, dose and frequency of virtual care offered to self-isolating COVID-19 patients. We identify the outcomes that arose from interventions delivered using virtual care among community-based COVID-19 patients. Finally, we identify areas that warrant further study in relation to the use of virtual care for intervention delivery among community-based patients with active COVID-19. 

## 2. Materials and Methods

We followed the scoping review methods recommended by the Joanna Briggs Institute [6]. Although not a systematic review, we sought to provide added structure to our scoping review process. We additionally followed the Preferred Reporting Items for Systematic Reviews and Meta-Analyses (PRISMA-ScR) scoping review checklist [7] including the development of a framework to guide scoping review question development, a comprehensive search strategy, screening using two independent reviewers, data extraction and analysis. We developed our scoping review questions using the population, concepts and context (PCC) framework and we have followed this format to guide development of eligibility criteria [8]. Using the PCC framework [7,8] outlined in Table 1, we identified the following scoping review question: “*What are the outcomes related to virtual care (telehealth and other modalities) provided to community-based COVID-19 patients in relation to the: (a) assessment and care provided during the self-isolation period, (b) use of technology for the delivery of patient care, and (c) self-reported patient experiences with virtual care*?”.

This scoping review considered all published empirical literature, including systematic reviews, meta-analyses, randomized trials, analytical cohort studies and cross-sectional studies. Qualitative studies were considered, including but not limited to designs such as phenomenology, grounded theory, ethnography, and qualitative design. We conducted searches across four bibliographic databases (CINAHL, Medline, Emcare, and Cochrane Database of Systematic Reviews). A sample of our search strategy is included as a Supplementary Materials. For this scoping review, we created a search strategy with consultation from a health sciences librarian. We specifically limited the date range of the publications to between the years 2020 and 2022 as COVID-19 is novel. Various combinations of subject headings and keywords were used; these included: COVID-19, COVID19, Novel Coronavirus, Coronavirus, SARS-COV-2, 2019-ncov, cov-19, virtual care, telemedicine, telehealth, telenursing, telepractice, telecare, telemonitoring, telecare, mhealth, ehealth, digital health, remote monitoring, remote consultation, ambulatory monitoring, home monitoring, isolation, quarantine, positive (COVID-19 positive), diagnosed and diagnosis. To account for variations of keywords that represent the concepts of interest, the Boolean operator OR was used. We adapted the use of keywords according to each of the four databases as needed, but all searches followed a combination of the use of the terms of virtual care AND COVID-19 AND quarantine AND positive. Initial practice searches resulted in a large number of records that discussed virtual care in the context of the COVID-19 pandemic rather than about virtual COVID-19 care. Our focus for this scoping review was not to understand virtual care in the era of COVID-19, but rather to focus specifically on virtual care use among COVID-19-positive patients who were instructed to quarantine or self-isolate at home in the community. After testing our search strategy, we decided to add the term “positive” as this addition yielded an increased proportion of records that were about virtual care for patients with COVID-19 and a decreased proportion of records that addressed the shifting to virtual modality during the pandemic in general.

We also conducted a search for potentially relevant literature in Google Scholar using the same search terms. We scanned the first 16 pages of Google output for potentially relevant literature. Any citations that were deemed relevant and fell within our years of search December 2020 to February 2022, we entered the citation into the eligibility screening process beginning with Level 1. 

DistillerSR software was used to manage the scoping review process. After implementing the search across databases, all abstracts and citations were uploaded into DistillerSR and duplicates removed. We used Distiller SR to manage the different levels of screening for the scoping review, including the creation of forms to input our responses for whether or not a citation was eligible for screening at the next stage. For this scoping review, we conducted two levels of eligibility screening. Level 1 assessed titles and abstracts as seen in Table 2. We used Level 1 screening as a way to review titles and abstracts for a clear focus on diagnosed COVID-19 patients (that is, those patients who were positive with COVID-19) who were carrying out self-isolation in the community and were not hospitalized during this period. We also included titles and abstracts that specifically referred to the use of virtual care as an intervention modality for the purposes of providing care for community-based self-isolating COVID-19 patients. 

During Level 2 screening, we examined full-text articles for those abstracts included from Level 1 as seen in Table 3. For Level 2 screening, we assessed full-text articles to ensure that they were empirical studies as opposed to theoretical or discussion papers that recommended virtual care but did not assess particular use of virtual care. We included full-text articles that focused on the delivery of health care through the virtual modality as a primary focus of the paper as opposed to articles that focused on virtual care used for COVID-19 screening, contact tracing, community surveillance, quarantine compliance or for the purposes of research data collection only. Finally, we included full-text articles that focused on COVID-19 patients in the active phase of disease as opposed to potential cases of COVID-19 not yet diagnosed, or cases where COVID-19 was resolved.

When full-text articles were included after Level 2 screening, we conducted handsearching of all reference lists. Any relevant citations were entered into the screening process beginning with Level 1 screening. All screening was conducted independently by two reviewers (L.C.-L., C.C.). All conflicts were discussed and resolved through discussion. Conflict resolution for each screening level involved re-reviewing the title, abstract and/or full article and examining the rationale for the reviewer’s selection and agreeing on a final decision. If a resolution could not occur, then a third person would be used to help resolve conflicts.

We excluded studies that were: (1) nonempirical studies, (2) case presentations (*n* = 1 or 2), (3) hypothetical or theoretical discussion of virtual care rather than evaluation of a virtual care intervention, and/or (4) focused on health providers or caregivers instead of the COVID-19 patients themselves. Screening criteria was strictly adhered to from the perspective of caring for persons with a COVID-19 diagnosis, as many articles demonstrated how virtual platforms assisted with surveillance [9], diagnosis [10] and contact tracing [11]. 

Data extraction was completed using a tool created specifically for this scoping review, to included information on the variables to help answer the review’s research questions and reach the review’s objectives Supplementary Materials. Data were extracted by one reviewer (L.C.-L.) and then verified by the second reviewer (C.C.). The data extracted included specific details about the included studies, the nature and delivery of the virtual care interventions and the outcomes resulting from virtual care administered to community-based self-isolating COVID-19 patients. Our analysis focused on “mapping the literature” and we analyzed extracted information for theoretical understanding of our concepts of interest. 

## 3. Results

Across the four bibliographic databases, 776 titles and abstracts were screened in Level 1 screening with 90 proceeding to Level 2 screening. During Level 2 screening, we screened 90 full-text articles with 19 articles being included into the scoping review. We excluded 757 citations. Inter-rater reliability across the two screeners for Level 1 initially began low (weighted Kappa 0.43) and improved after discussion of conflicts with the highest level of inter-rater reliability for our first screening question (weighted Kappa 0.75). Inter-rater reliability was consistent across all eligibility questions in Level 2 screening with an overall weighted Kappa score of 0.53. Again, all conflicts arising through this screening stage were discussed and resolved among the two screeners. While we would have liked to have seen higher inter-rater reliability, these results were consistent with the complexity of the literature. 

## 4. Discussion and Future Research

The full results from this scoping review will be reported elsewhere [12]. Using the scoping review methods allowed us to contextualize and highlight emerging insights regarding the use of various virtual care modalities for community-based, self-isolating COVID-19 patients. Because the pandemic has been a unique situation, many healthcare organizations likely struggled with implementing formalized mechanisms for follow-up for these types of patients. For example, patients might be discharged from hospital and may have been told to self-isolate, but primary care services or public health may not have been notified in time of their situation. Likewise, newly diagnosed COVID-19 patients who are self-isolating may not have access to primary care resources making outreach virtual care initiatives essential. From the literature that was included in this review, many hospitals or ambulatory care clinics made attempts to follow this group of patients by offering follow-up virtual visits, creating virtual wards, or referring positive COVID-19 patients to specialty clinics for follow-up during the isolation period. This type of follow-up would not likely occur for a typical hospital discharge visit and, in some cases, patients were referred to primary care. Healthcare providers and organizations clearly worked carefully and rapidly to create a virtual care “safety” network for isolating patients in the community. Future research might examine the provision of this type of seamless care—from hospital to home using virtual care as the mechanism for follow-up and linkage. 

In addition to research that investigates how well targeted virtual care initiatives work in terms of provision of seamless transition from hospital to community, continued research is needed to better understand how virtual care should be administered (e.g., timing and dose) to be most useful to isolating COVID-19 patients. Our scoping review identified virtual care programming that varied in the type of virtual methods used, varied in the duration of virtual services offered and varied in terms of the clinical outcomes being investigated. Future research could consider virtual care as an intervention and examine its effectiveness in addressing key identified outcomes specific to the COVID-19 population.

Finally, our results indicated that we had a high level of disagreement initially as independent screeners, despite fairly detailed eligibility criteria for inclusion. We estimate that this was due to the fact that the literature was quite diverse with many studies written by front-line clinicians who were reporting on new virtual care initiatives or programs or “emergency” use of virtual care to manage COVID-19 patients. Much of what we saw in the literature indicated that virtual care initiatives were conducted that were not part of pre-planned research studies. These were more likely to be initiatives quickly implemented as a response to the current health care situation. However, we aimed to follow the iterative nature for conducting scoping reviews, and therefore continuously improved the screening process together. We encountered literature that was written in a variety of formats where there was inconsistency in how virtual care as a modality was administered and used for COVID-19 patients. We considered the use of a systematic review method but decided against it because the large proportion of literature would be critically appraised as poor quality due to issues mentioned earlier. There was also a need to map out the concepts relevant to community-based COVID-19 virtual care rather than evaluating the efficacy of the virtual care interventions. We felt there was merit to use a scoping review method to describe and present the context for what occurred in terms of virtual care delivery during the height of the COVID-19 pandemic. Clearly, future research could include the evaluation of various approaches to virtual care among COVID-19 patients who are recently diagnosed in the community.

## 5. Conclusions

The results from this scoping review offered a description of and a map for the available evidence regarding virtual as a modality to offer various interventions for community-based self-isolating patients with active COVID-19. Identified gaps in the literature were discussed. Key outcomes arising from the use of various virtual care modalities were assessed as part of this scoping review, including the various types and usage of virtual care among COVID-19 patients.

## Figures and Tables

**Table 1:** Population, Concept and Context (PCC) framework.

Framework Component	Criteria
Population	COVID-19 patients (non-hospitalized, outpatient, self-isolating, community) receiving any healthcare intervention that is delivered by virtual modality
Concepts	Increased follow-up, support, assessment during self-isolation, increased compliance with COVID-19 isolation requirements
Context	Increased use of technology for delivery of care; Consideration of multiple forms of virtual care including telehealth, video conferencing, smart apps, wearable technology or other types of virtual care; Care delivered for the purpose of treating COVID-19 patients who were isolating in place

**Table 2:** Level 1 Eligibility Screening Questions.

Question Number	Question	Answer
1	Does the title or abstract address the community-based self-isolating COVID-19 patient population (COVID-19 patient includes a diagnosis or positive result)? Exclude: -epidemiological surveillance -contact tracing/case tracking, symptom or case monitoring for purpose of quarantine compliance (that is, not providing care) -case presentation/report; descriptions of one or two COVID-19 patients (*n* = 1)	Yes—Include No—Exclude Cannot tell—Include
2	Does the title or abstract address the use of virtual care for the purpose of caring for the community-based self-isolating COVID-19 patient (virtual care includes telemedicine, telehealth, mhealth, ehealth, digital health, remote monitoring, remote consultation)? Exclude: -if comorbid condition, e.g., diabetes, but the virtual care is for diabetes management, not for COVID -talk about technologies/virtual care hypothetically but not administering the virtual care to a COVID-19 patient	Yes—Include No—Exclude Cannot tell—Include
3	Does the title or abstract indicate outpatient quarantine (in the community or at home, that is, not in-hospital)? Include: -isolation centres/hotels Exclude: -long-term care homes	Yes—Include No—Exclude Cannot tell—Include

**Table 3:** Level 2 Eligibility Screening Questions.

Question Number	Question	Answer
1	Does the article describe an empirical (research) study? Include: All quantitative or qualitative research studies. Included also are systematic reviews, meta-analyses and literature reviews with a core focus on virtual care for community-based self-isolating COVID-19 patients. Exclude: case presentation/report; descriptions of one or two COVID-19 patients (*n* = 1 or 2) -discussion/opinion/reflective/editorial pape r-conference papers -non-peer-reviewed articles -articles that recommend how virtual care could be used for COVID-19 patients but do not implement -best practice guidelines/procedural pape r-study protocol where the research has not occurred yet -systematic reviews, meta-analyses, literature reviews that focus only on virtual care and not related to community-based COVID-19 patients	Yes—Include No—Exclude Cannot tell—Include
2	Does the study describe the delivery of health interventions to COVID-19 patients, where the primary intervention uses virtual modality (that is planned/intentional use of virtual care; intention to use virtual care for COVID-19 patient as opposed to “pivoted” to virtual care)? Does the results section include analysis for the virtual care component and is it focused on the COVID 19 patient (as opposed to analysis on other care providers; family members etc.)? Exclude: offering virtual care as an offshoot using technology but not to deliver care -improving/evaluating health provider training -implementing virtual care as a department/organization in response to pandemic restrictions -epidemiological surveillance or contact tracing/case tracking, symptom or case monitoring for purpose of quarantine compliance (that is, not providing care) -use of technology to screen for COVID before entering a facility talk about technologies/virtual care hypothetically as a good recommendation but not administering the virtual care to a COVID-19 patient -social media that does not include any interaction between care provider and patient (that is, general messaging accessible to the public rather than customized for the individual patient) -use of virtual modality for the purpose of data collection for the study rather than delivery of care	Yes—Include No—Exclude Cannot tell—Include
3	Is the study population of diagnosed COVID-19 patients during the active phase of the virus/illness (e.g., recently diagnosed, in self-isolation or quarantining during the period of communicability) in the community (that is, not in-person/inpatient at a health care facility)? Include: -studies with patients in the home or in the community -studies with COVID hotel or isolation centres Exclude: -studies that look at potential cases of COVID-19 where the patient population has not been assessed for or diagnosed with COVID-19 -studies where COVID-19 has been resolved and patients are healthy	Yes—Include No—Exclude Cannot tell—Include

## Data Availability

Not applicable.

## Supplementary Materials

The following supporting information can be downloaded at: https://www.mdpi.com/article/10.3390/healthcare10101847/s1, Data Extraction Tool.
