# Supplementary material for: How Has COVID-19 Changed the Way We Do Virtual Care? A Scoping Review Protocol
Source: Healthcare (Basel). 2022 Sep 23;10(10):1847. doi: 10.3390/healthcare10101847 (PMC9601957; doi:10.3390/healthcare10101847)
Supplement: Supplementary file 1 [file healthcare-10-01847-s001.zip › healthcare-1852700-supplementary.pdf]

Data Extraction Tool

**Author(s)**	**Year**	**Country**	**Purpose Statement**	**Design**	**Sample and patient characteristics**<br><br>**Any power analysis? Y/N**	**Variables**					**Implications for Research, Practice, and Policy**
						**Types of health service and supports**	**Technology used to deliver virtual care (what and how)**	**Who provides the virtual care?**	**Patient experience**	**Patient outcomes**	
